# Heat extraction performance of supercritical carbon dioxide flow in rough fractured rock

**DOI:** 10.1016/j.isci.2026.115131

**Published:** 2026-02-24

**Authors:** Xiao Xiaochun, Li Cenrui, Gong Peiyushun

**Affiliations:** 1 School of Mechanics and Engineering, Liaoning Technical University, Fuxin 123000, China

**Keywords:** materials science, Computational materials science, modeling in materials science

## Abstract

This study proposes a parameterized approach to construct three-dimensional rough fracture surfaces by superimposing cosine functions with adjustable spatial frequencies and random phase distributions. Numerical simulations are performed to investigate heat extraction from hot dry rock using supercritical carbon dioxide (ScCO_2_), with particular emphasis on the coupled effects of non-Darcy flow and fracture structural characteristics. The results indicate that fracture dip angle plays a critical role in heat extraction efficiency by influencing the stability of the pressure gradient. When the fracture baseline forms a 30° dip angle with the injection-production well line, rough fractures facilitate stable unidirectional seepage between wells, achieving a heat extraction power of 5.5 MW, which is significantly higher than that at 0° and 60°. In addition, increasing fracture roughness enhances flow tortuosity and alters seepage dynamics, thereby affecting the overall heat extraction performance.

## Highlights

• Parameterized modeling of 3D rough fracture surfaces
• Fracture dip angle controls ScCO_2_ seepage stability
• Optimal dip angle maximizes heat extraction
• Low fracture roughness improves flow and heat recovery

## Graphical abstract

**Figure:**
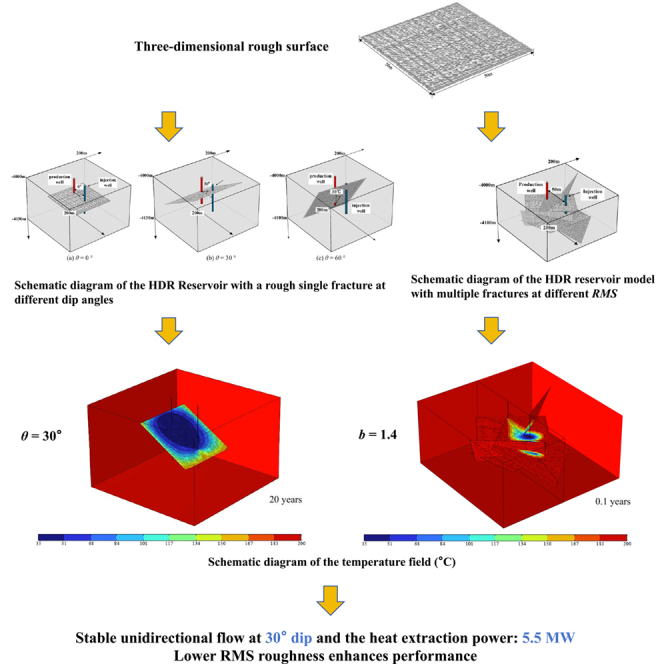

## Introduction

Amid the global transition to a cleaner energy structure, hot dry rock (HDR) geothermal resources have emerged as a key focus for new energy development due to their abundant reserves and zero-carbon emission characteristics.^1^ As the primary engineering means for extracting HDR resources, enhanced geothermal systems (EGSs) have traditionally relied on water as the heat transfer fluid. However, this conventional approach faces inherent challenges such as premature thermal breakthrough, limited reservoir longevity, and the risk of inducing seismic activity.^2^ The use of supercritical carbon dioxide (ScCO_2_) as the heat exchange fluid for extracting HDR geothermal resources holds the potential to effectively address these issues.^3^ However, predicting the behavior of ScCO_2_ in fractured reservoirs is fundamentally challenging. Natural rock fractures often exhibit multi-scale roughness and complex three-dimensional geometries. Additionally, fluids flowing through these fractures typically display significant non-Darcy seepage characteristics at high velocities. Understanding the coupling mechanism between fracture geometry and non-Darcy flow is crucial for optimizing ScCO_2_-EGS system performance.^4^

Snow^5^ proposed the cubic law based on the smooth parallel plate assumption, stating that the fluid flow rate in a fracture is proportional to the cube of the fracture aperture. While this model has been widely applied in fields such as groundwater flow, geothermal development, and nuclear waste disposal,^6^ it fails to account for the roughness and uneven aperture distributions found in natural fractures, leading to significant deviations from real-world flow behavior. For example, Tsang^7^ demonstrated through numerical simulations that the equivalent permeability of rough fractures is only 1/10 to 1/5 of that predicted by the parallel plate model. Physical experiments by Brown,^8^^,^^9^ and granite fracture seepage tests by Wang et al.^10^ have confirmed that the flow rate in rough fractures can deviate by 1–2 orders of magnitude from the cubic law’s predictions. Recent high-resolution flow experiments^11^ have further corroborated these findings. To address these limitations, the concept of equivalent hydraulic aperture was introduced. Lomize^12^ proposed an empirical formula to convert fracture surface roughness into an equivalent hydraulic aperture. Louis^13^ introduced a contact area ratio correction model, highlighting the significant effect of tortuosity on permeability. Long et al.^14^ established a relationship between the fractal dimension of fracture roughness and the equivalent hydraulic aperture. However, this model still relies on idealized fractal parameters, which limit its application to complex natural fractures. Most existing correction models focus on a single surface morphology parameter, failing to capture the full complexity of fracture geometry. For example, Zhang et al.^15^ used 3D printing technology to reveal that local protrusions and depressions on fracture surfaces significantly increase flow resistance—an effect not accounted for in traditional models.

As the flow velocity increases, the fluid flow in fractured rock masses gradually exhibits distinct nonlinear characteristics. Zimmerman et al.^16^ observed experimentally that when the Reynolds number *Re* > 10, the pressure drop is related to the square of the flow velocity. This phenomenon is especially pronounced in rough fractures. Numerical simulations by Xu et al.^17^ suggest that the concave-convex structures on fracture surfaces can induce local eddies, thereby exacerbating the inertial resistance effect. The critical Reynolds number is a crucial parameter for determining whether seepage enters the non-Darcy regime, but its value is influenced by multiple factors, including fracture geometry, stress state, and fluid properties, which leads to significant discrepancies in the results of different studies. Zhang et al.^18^ and Chen et al.^19^ have demonstrated that both shear displacement (leading to mismatched fractures) and increased normal stress can significantly reduce the critical Reynolds number, promoting the earlier onset of non-Darcy flow by creating more tortuous flow paths. Zhang et al.^20^ constructed a three-dimensional rough single-fracture rock model and found that an increase in normal stress leads to a significant change in the critical pressure gradient. Xiong et al.^21^ employed 3D printing technology to replicate natural rough fractures and found that a 20% increase in root-mean-square roughness (*RMS*) reduced the critical Reynolds number by 30%, further confirming the enhancing effect of fracture roughness on non-Darcy flow. Deng et al.^22^ proposed a 2D model that incorporates fracture *RMS*, roughness index, and correlation length, unifying the normal stiffness-permeability relationship for fractures under both the effective medium and percolation regimes. Adler et al.^23^ observed through microfluidic experiments that fluid is predominantly concentrated in a few high-aperture channels, while low-aperture regions scarcely participate in flow. Pyrak-Nolte^24^ further emphasized that the presence of preferential flow significantly reduces the effective permeability of fractures and causes a sharp increase in nonlinear pressure drop. Wavelet analysis indicates that first-order roughness governs the primary fluid flow direction, while second-order roughness further exacerbates non-Darcy flow by forming local eddies or backflows.^25^^,^^26^

Existing research has generally overlooked the impact of non-Darcy flow in HDR resource extraction and its relationship with three-dimensional fracture structural characteristics. This study generates three-dimensional fracture surfaces with varying roughness by superimposing cosine functions with different spatial frequencies and random phases. It quantifies how structural features, such as dip angle and roughness, influence ScCO_2_ seepage direction, pressure gradient, and heat exchange area. This research reveals the relationship between ScCO_2_ flow evolution in three-dimensional rough fractures and EGS heat exchange efficiency, providing a foundation for three-dimensional spatial optimization in reservoir fracturing modification.

## Results and discussion

This study combines numerical simulation and theoretical analysis. The simulation provides insights into the system’s behavior, while the theoretical analysis reveals the underlying principles, ensuring the comprehensiveness and reliability of the results.^27^

### Model assumptions

The lithology of the HDR reservoir is primarily acidic granite,^28^ and the deep formation is typically water-free, thus chemical reactions are neglected. This article makes the following assumptions.

**Figure 1:**
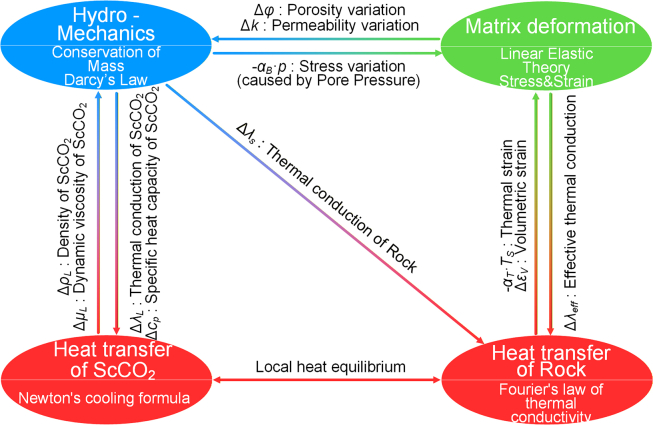
Schematic diagram of the coupling relationships in the THM model

• This study does not address the processes of rock damage or fracture propagation. Therefore, the rock matrix is modeled as a homogeneous, isotropic, linear elastic material to simplify the computations;
• Fluid flow in the matrix is saturated single-phase laminar flow, conforming to Darcy’s law; flow in fractures is non-Darcy flow, satisfying Forchheimer’s law, with mass exchange between them represented by source terms;
• The temperature field employs third-type boundary conditions, and heat exchange between the matrix and fractures is represented by heat source terms;
• The CO_2_ working fluid remains in a supercritical state;
• Due to the large scale and long operational duration of EGS, ScCO_2_ achieves sufficient heat exchange with the hot dry rock reservoir. Therefore, the local thermal equilibrium model is adopted to describe the heat transfer process;
• Within the range of fracture flow and temperatures investigated in this study, heat conduction and convection are the dominant heat transfer mechanisms; therefore, thermal radiation is neglected;
• The inertial forces associated with the deformation of reservoir rocks are neglected.
• Fractures do not propagate during the extraction process. The system coupling relationships is shown in Figure 1.

### Model description

#### Description of three-dimensional rough fracture surfaces

*RMS* is a quantitative parameter based on the statistical distribution of surface heights and is widely used to characterize the roughness of material surfaces.^29^ In rock mechanics, the *RMS* value quantifies the amplitude characteristics of surface undulations by calculating the square root of the mean square of the deviations between the fracture surface heights and a reference plane. Compared to the Joint Roughness Coefficient (*JRC*), the *RMS* value directly quantifies roughness through mathematical calculation, thus avoiding errors introduced by human judgment. Furthermore, since large-scale fractures typically exhibit significant macro-undulations, the *RMS* value can effectively reflect these large-scale height variations.

The three-dimensional fracture model provides a more realistic representation of natural fracture morphology by incorporating surface roughness and aperture heterogeneity, enabling the capture of tortuous flow paths and local eddies that cannot be resolved in two-dimensional models. Fracture surfaces were generated using the spectral superposition method described in the STAR Methods, in which the spectral exponent *b* controls the attenuation of high-frequency components in the surface height field. Physically, *b* governs the relative contribution of small-scale asperities: larger *b* values suppress high-frequency components and produce smoother fracture surfaces, whereas smaller *b* values enhance fine-scale roughness.

**Figure 2:**
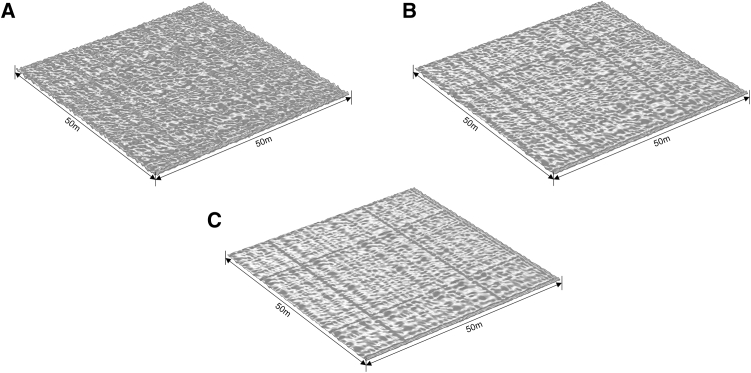
Schematic diagram of the different rough fracture surfaces (A) *b* = 1.4. (B) *b* = 1.6. (C) *b* = 1.8.

By setting *b* = 1.4, 1.6, and 1.8, three representative roughness levels were constructed (Figure 2). The *RMS* roughness was calculated as described in the STAR Methods. The corresponding *RMS* values are 1.1093 m, 1.0043 m, and 0.9413 m, representing high-, medium-, and low-roughness fractures, respectively.

### Establishment of the geometric model

**Figure 3:**
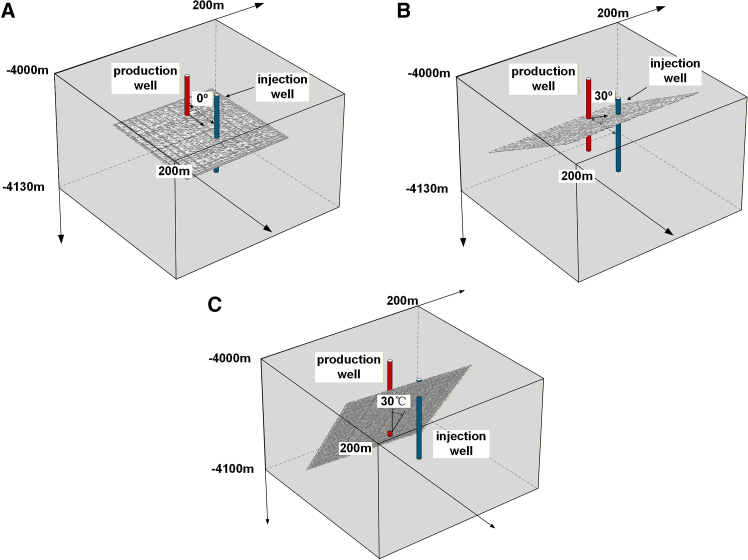
Schematic diagram of the HDR reservoir with a rough single fracture at different dip angles (A) *θ* = 0°. (B) *θ* = 30°. (C) *θ* = 60°.

Assuming that the HDR reservoir is buried at a depth of 4000 m, with an initial reservoir temperature of 200 °C, the geometric model of the reservoir is a rectangular cuboid with dimensions of 200 m × 200 m × 130 m. The wellbore diameter is 0.26 m, and the spacing between the injection and production wells is 50 m. The blue cylinder represents the injection well, and the red cylinder represents the production well, with the fracture located between the two wells. By selecting the angle between the baseline of the rough fracture and the line connecting the injection and production wells as a variable, a single-fracture heat extraction model for the HDR reservoir with different dip angles was established. Three geometric models of the reservoir with dip angles *θ* of 0°, 30°, and 60° were created, and the injection well and production well penetrated the rough fracture surface in all models, as shown in Figure 3. The fracture surface is a medium-roughness surface with an *RMS* value of 1.0043 m when *b* = 1.6, and the “length × width” of the fracture surface is 50 m × 50 m.

**Figure 4:**
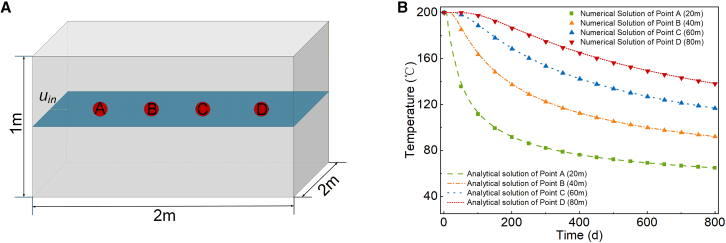
Schematic diagram of the THM coupling verification model (A) Verification model diagram. (B) Comparison of numerical and analytical solutions.

The specific parameter values of the model are shown in Table 1.^30^

**Table 1:** Basic parameters of the 3D HDR reservoir numerical simulation

Model Parameter	Value	Unit
Rock matrix specific heat capacity *c*_*S*_	1000	J/(kg·°C)
Rock matrix thermal conductivity *λ*_*S*_	3	W/(m·°C)
Rock matrix density *ρ*_*S*_	2700	kg/m^3^
Rock matrix elastic modulus *E*	55	GPa
Rock matrix Poisson’s ratio *υ*	0.25	1
Rock matrix bulk modulus *K*_*S*_	54.5	GPa
Rock matrix thermal expansion coefficient *α*_*T*_	3 × 10^−6^	1/K
Injection well pressure *p*_in_	12	MPa
Production well pressure *p*_out_	10	MPa
Initial pore pressure *p*_0_	10	MPa
Initial rock matrix porosity *ϕ*_*S*0_	0.02	1
Initial rock matrix permeability *k*_*S*0_	10^−18^	m^2^
Initial fracture aperture *d_f_*	1	mm
Fracture porosity *ϕ*_*f*_	1	1
Initial fracture permeability *k*_*f*0_	8 × 10^−6^	m^2^
Dimensionless friction coefficient *c*_*F*_	1.75/(150*ϕ*_*f*_^3^)^1/2^	1
Forchheimer coefficient *β*	*c*_*F*_/(*k*_*f*_)^1/2^	1/m
Fracture Biot coefficient *a*	1	1
Fracture normalization constant *b*_1_	2 × 10^−7^	1/Pa
Roughness crack surface profile index *b*	1.6	1
Spatial resolution *N*	20	1
Range of the spatial coordinate *x* (-*s*_1_, *s*_1_)	(-50, 50)	m
Range of the spatial coordinate *y* (-*s*_2_, *s*_2_)	(-50, 50)	m

**Table 2:** Basic parameters of the verification model

Model Parameter	Value	Unit
Rock matrix specific heat capacity *c*_*R*_	1000	J/(kg·K)
Reservoir matrix thermal conductivity *λ*_*S*_	2.8	W/(m·K)
Reservoir matrix density *ρ*_*R*_	2700	kg/m^3^
Specific heat capacity of working fluid *c*_*w*_	4200	J/(kg·K)
Density of working fluid *ρ*_*w*_	1000	kg/m^3^
Dynamic viscosity of working fluid *μ*_*w*_	0.001	Pa·s
Injected fluid velocity *u*_in_	0.02	m/s
Initial rock temperature *T*_*i*_	200	°C
Injected fluid temperature *T*_in_	35	°C
Fracture aperture *d*_*fr*_	1	mm
Fracture inertia resistance coefficient *β*	495.07	1/m
Dimensionless friction coefficient *c*_*F*_	0.14289	1

Related model validation is shown in Figure 4, and the specific validation method and model parameters (Table 2) are detailed in STAR★Methods.

### Comparative analysis of heat recovery performance of supercritical carbon dioxide in reservoirs with different fracture inclinations

**Figure 5:**
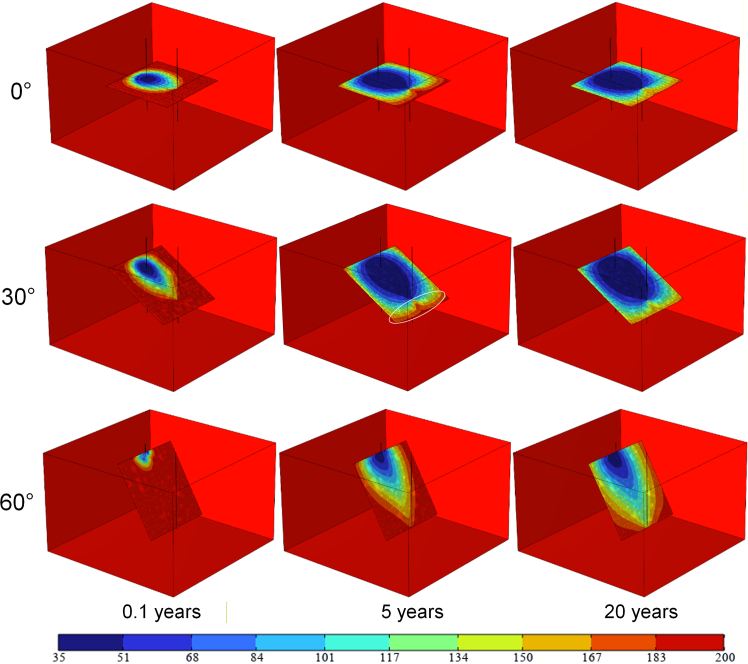
Temperature field of the HDR reservoir with a rough single fracture at different dip angles (°C)

As shown in Figure 5, the temperature field results for coarse single fractures with dip angles of 0° and 30° indicate that ScCO_2_ rapidly reaches the vicinity of the production well during the initial system operation phase. The fracture surfaces connecting the two wells establish efficient fluid flow pathways within the reservoir. Compared to the temperature field of the HDR reservoir at 0° dip angle, the low-temperature zone at 30° dip angle exhibits a sharper front edge and less diffusion. The 30° dip angle of the fracture concentrates the heat exchange area of ScCO_2_ more around the line connecting the two wells, enhancing the heat extraction efficiency of ScCO_2_. The pressure difference between the injection and production wells creates a pressure gradient between them, with the greatest pressure drop occurring along the line connecting the two wells. The “tip” of the low-temperature zone’s edge reaches the production well first. Driven by this pressure gradient, the ScCO_2_ flow converges around the line connecting the injection and production wells. ScCO_2_ that crosses the production well must flow back against the pressure gradient to return to it. This process results in relatively weak heat exchange for the ScCO_2_, leading to the formation of a low-temperature zone that “envelops” the production well along the reverse flow pressure gradient.

When the rough single-fracture dip angle is 60°, the low-temperature zone formed on the fracture surface does not reach the production well during the initial stage of system operation. Furthermore, throughout the entire system operation period, the diffusion of the low-temperature zone is slower compared to fracture reservoirs with 0° and 30° dip angles, indicating that ScCO_2_ exhibits relatively weaker heat extraction performance during system operation. This indicates that compared to the 0° and 30° dip angle conditions, ScCO_2_ did not achieve sufficient heat exchange within the rough fracture at a 60° inclination.

**Figure 6:**
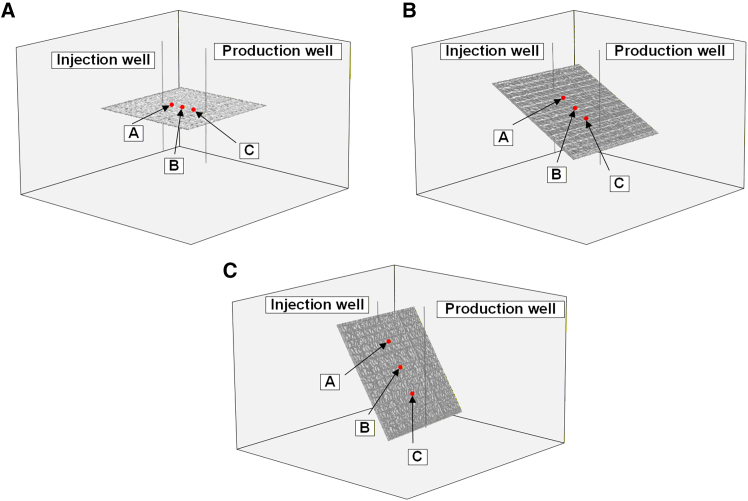
Schematic diagram of the distribution of monitoring points within fractures with different dip angles (A) *θ* = 0°. (B) *θ* = 30°. (C) *θ* = 60°.

To quantitatively describe its temporal variation, monitoring points were arranged in single fractures with different inclinations, as shown in Figure 6. In each of the three fracture inclinations, the monitoring points were spatially distributed at equal intervals: Probe Point A is located near the injection well; Probe Point B is positioned between the injection and production wells; Probe Point C is situated near the production well.

**Figure 7:**
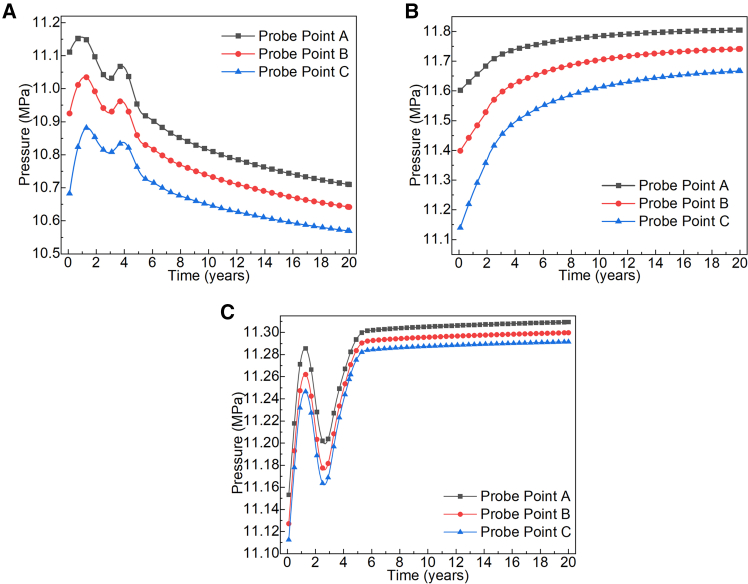
Pressure evolution at monitoring points across fractures with different dip angles (A) *θ* = 0°. (B) *θ* = 30°. (C) *θ* = 60°.

Figure 7 shows the pressure variation over time at various monitoring points within fractures of different inclinations. It can be observed that during the first five years of system operation, significant pressure fluctuations occurred at all monitoring points across fractures of varying inclinations. The differences in probe placement locations also resulted in distinct overall pressure levels. At this stage, since the flow pathways for ScCO_2_ within the fractures had not yet fully developed, the pressure differential between injection and production drove rapid ScCO_2_ filling throughout the fractures, increasing its flow velocity. Under the influence of non-Darcy flow, the inertial resistance of ScCO_2_ flow within the fractures generated localized oscillations. During the first 5–10 years of system operation, the pressure gradient within the fractures rapidly stabilized. This indicates that over this period, the progressively enhanced permeability of the fractures gradually stabilized the overall flow direction of ScCO_2_. Due to the rough surface morphology of the fractures, the local flow paths of ScCO_2_ are non-uniform. Under the combined effects of injection-production pressure differentials and non-Darcy flow, the seepage pressure in 0° dip angle fractures gradually exhibits a nonlinear decline. In contrast, for 30° and 60° dip angle fractures, the elongation of flow pathways leads to ScCO_2_ velocity gradually stabilizing with continued system operation. This ultimately establishes a stable pressure gradient within the fractures, sustaining consistent ScCO_2_ mass transfer.

By observing the average seepage pressures across the three dip angles, it is evident that the average seepage pressure at the 30° dip angle is higher than at the other two dip angles. The primary reason for this difference is that stable ScCO_2_ convective heat and mass transfer within the fracture accelerates diffusion in the fracture’s low-temperature zone. This rapidly lowers the temperature of the surrounding rock matrix, causing it to gradually “contract.” Consequently, the fracture opening progressively increases, enhancing permeability and reducing flow resistance for ScCO_2_. Under conditions of continuously increasing flow velocity and intensified non-Darcy flow, the flow direction of ScCO_2_ within the 30° dip angle fracture exhibits no significant fluctuation. The 60° dip angle fracture plane forms a relatively large angle with the direction of the maximum principal stress in the reservoir, increasing the normal stress component acting on the fracture. This enhances the fracture’s tendency to close and combined with the cooling contraction of the rock matrix, the hydraulic forces reduce the extent of fracture opening. This makes it difficult for the fracture to enhance the permeability of ScCO_2_ and form effective ScCO_2_ flow pathways.

**Figure 8:**
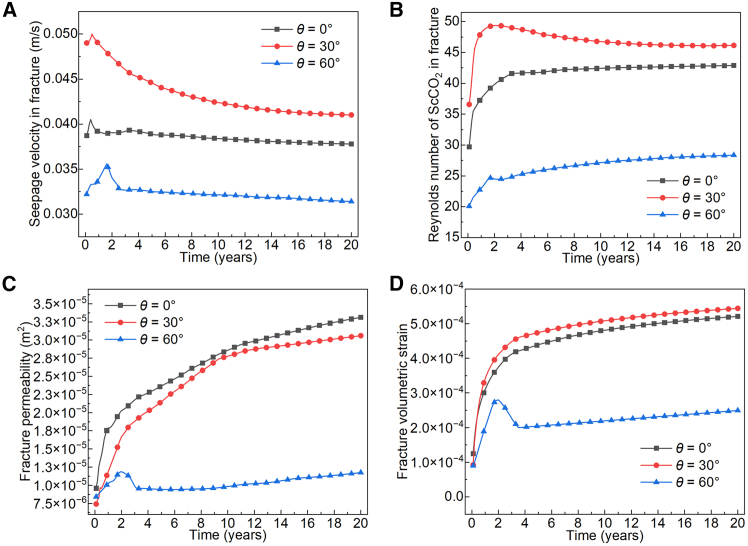
Flow characteristics of ScCO_2_ in rough fractures with different dip angles (A) Average seepage velocity in fractures. (B) Reynolds number of ScCO_2_ in fractures. (C) Fracture permeability. (D) Fracture volumetric strain.

Figure 8 shows that ScCO_2_ flow in fractures with different dip angles exhibits non-Darcy behavior (Reynolds number *Re* > 10); hence, inertial forces cannot be neglected. During system operation, the seepage velocities of ScCO_2_ in fractures with different dip angles display three distinct patterns, all characterized by a brief initial increase, followed by a decrease, and finally stabilization. The stabilization process of the pressure gradient within the fractures aligns with the trend of ScCO_2_ seepage velocity: for a 0° dip angle, pressure-gradient stabilization involves repeated adjustments between the two wells, consistent with the slight fluctuations in ScCO_2_ seepage velocity during the first five years; for a 30° dip angle, pressure-gradient stabilization proceeds monotonically from the injection to the production well, matching the monotonic variation in ScCO_2_ seepage velocity; for a 60° dip angle, the seepage pressure equilibrates rapidly within the first five years, and no stable pressure gradient is established by the end of the simulation, which again corresponds to the observation that ScCO_2_ seepage velocity shows no significant change after year 5. Note that the seepage velocity is defined as the spatial average of speed magnitude over the fracture, irrespective of flow direction.

As indicated by the Reynolds number of ScCO_2_ flow in Figure 8B, non-Darcy flow affects the stabilization of the pressure gradient in the fracture. At the beginning of system operation, ScCO_2_ seepage velocity increases under the injection-production pressure difference. Subsequently, non-Darcy effects strengthen, inertial resistance rises relative to viscous resistance, and the seepage velocity decreases. This change in velocity influences mass transfer of ScCO_2_ in the fracture, alters the volume of ScCO_2_ present, and thereby affects pressure-gradient stabilization. After about 10 years, non-Darcy flow gradually stabilizes, its influence on ScCO_2_ flow diminishes, seepage velocity approaches a steady value, and a stable pressure gradient gradually forms in the fracture.

During system operation, the permeability of fractures with different dip angles gradually increases (Figure 8C). Based on the fracture volumetric strain maps at different dip angles (Figure 8D), fractures undergo rapid dilation under hydraulic pressure in the early stages. However, the dilation increase is relatively smaller for fractures with a 60° dip angle due to the higher *in-situ* stress and steep inclination. Although the ScCO_2_ seepage velocity in the 60°-dip fracture rises rapidly in the first two years, between years 2 and 3, the aperture gradually decreases under combined thermal contraction and *in-situ* stress, leading to a reduction in permeability. After year 10, the permeability increase in the 30° dip-angle fracture slows down. Nevertheless, because a favorable ScCO_2_ flow pathway and a stable pressure gradient have been established between the injection and production wells, the fracture maintains a steadily increasing aperture under the coupled effect of hydraulic, *in-situ*, and thermal stresses, so the average volumetric strain of the rough fracture continues to grow slowly. The permeability of the 0° dip-angle fracture remains close to and mostly higher than that of the 30° dip-angle fracture throughout the simulation, yet the ScCO_2_ seepage velocity in the 0° dip-angle fracture is lower. Since the seepage velocity reflects only speed magnitude, the stabilization process of the pressure gradient may have altered the flow direction of ScCO_2_ in the fracture.

**Figure 9:**
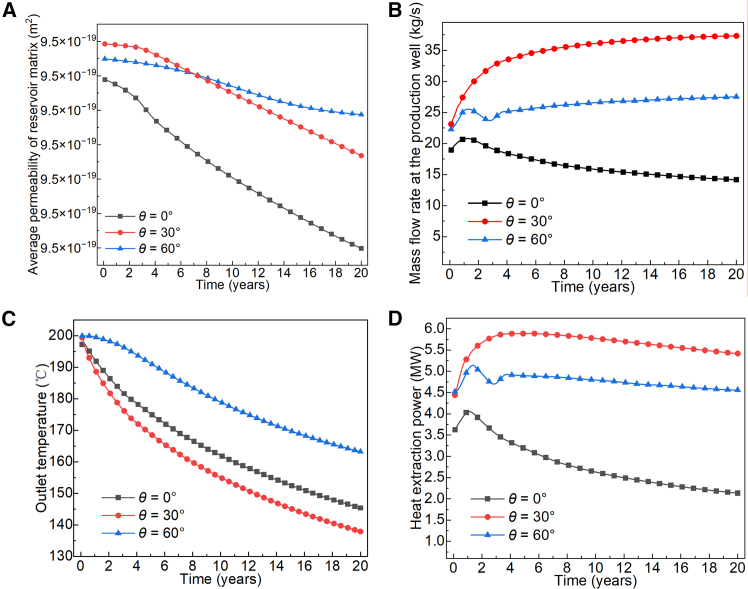
Heat extraction performance of ScCO_2_ in the EGS with rough fractures at different dip angles (A) Average permeability of reservoir matrix. (B) Mass flow rate at the production well. (C) Outlet temperature. (D) Heat extraction power.

As shown in Figure 9, the permeability of the rock matrix gradually decreases due to thermal contraction as the reservoir cools. Although the fracture permeability at a 60° dip angle is lower than that at 0° and 30°, the higher matrix permeability at this steep inclination allows ScCO_2_ mass transfer through the matrix to remain unaffected by earlier fluctuations in fracture permeability after year 3. In contrast, in fractures with 0° dip angle, the flow direction of ScCO_2_ shifts during the pressure gradient equilibration process, hindering mass transfer. This effect persists throughout the system operation, ultimately hindering ScCO_2_ recovery. The continuous injection of low-temperature ScCO_2_ into the high-temperature reservoir through the production well, combined with incomplete production, reduced thermal extraction performance. Smoother flow and more stable recovery in 60° dip-angle fractures promote uniform heat extraction across the reservoir, resulting in a higher average temperature at the production well and thus better thermal performance compared to the 0° dip-angle case.

Research findings indicate that fracture dip angle significantly influences the direction of seepage and the formation of pressure gradients. Fractures with a 30° dip angle exhibit stable unidirectional pressure gradients between the injection and production wells, enabling more consistent mass transfer of ScCO_2_ and achieving the highest average heat extraction efficiency (5.5 MW). This outperforms the repeated pressure equilibrium observed in 0° dip angle fractures and the seepage stagnation issues encountered in 60° dip angle fractures.

### Effect of crack roughness on heat transfer performance

The detailed algorithms for non-Darcy flow determination and the quantification of heat extraction efficiency are provided in the STAR Methods.

**Figure 10:**
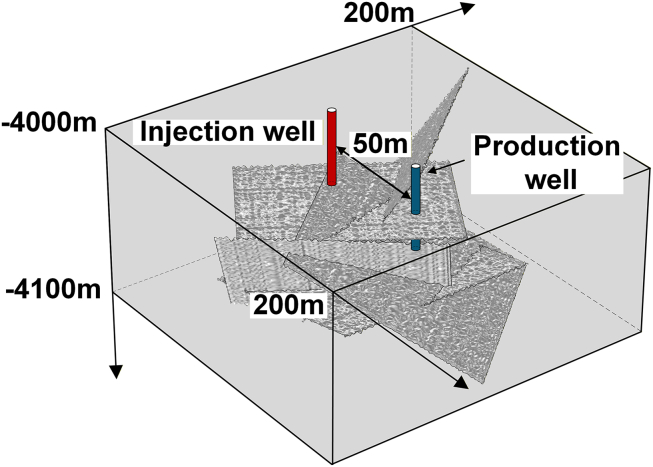
Schematic diagram of the HDR reservoir model with multiple fractures at different *RMS*

Based on the Monte Carlo method, generate five fractures with dip angles ranging from 0° to 60° within a rectangular prism region of dimensions 200 m × 200 m × 100 m. By keeping the fundamental parameters of the HDR reservoir geometric model constant and varying the *b* value to 1.4, 1.6, and 1.8, HDR reservoirs with different roughness crack surfaces can be obtained, yielding *RMS* values of 1.1093 m, 1.0043 m, and 0.9413 m, respectively. Using numerical integration methods, the surface areas of the three different *RMS* roughness fractures were calculated to be 52,783 m^2^, 50,832 m^2^, and 49,497 m^2^, respectively, as shown in Figure 10.

**Figure 11:**
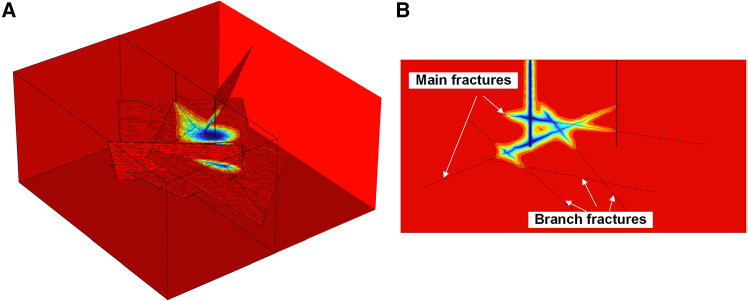
Schematic diagram of the HDR reservoir model with multiple fractures at different *RMS* (A) Temperature cloud map. (B) Schematic diagram of the main fracture and branch fractures.

To facilitate the analysis of thermal performance, the five coarse fractures were categorized into two types: main fractures and branch fractures. Main fractures refer to large-scale fractures that are directly connected to the injection and production wells and serve as the primary flow channels. Branch fractures, on the other hand, are not directly connected to the wells but form secondary structures through connections with the main fractures. Their role is to enhance system connectivity and flow complexity, rather than acting as primary channels for mass and heat transfer. For clarity, a cross-sectional view of the temperature contour map for a production well in a low-roughness fracture reservoir (0.1 years) is shown in Figure 11.

**Figure 12:**
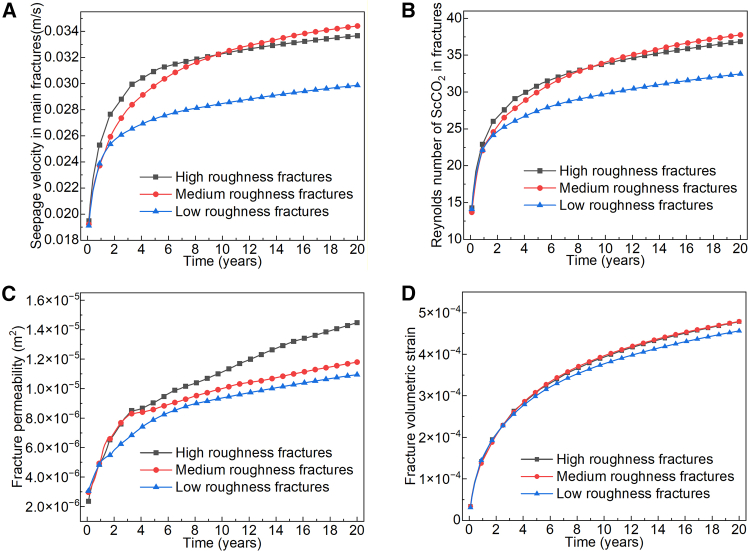
Flow characteristics of ScCO_2_ in fractures with different *RMS* (A) Seepage velocity in main fractures. (B) Reynolds number of ScCO_2_ in fractures. (C) Average permeability of fractures. (D) Fracture volumetric strain.

As shown in Figure 12, non-Darcy flow (*Re* > 10) occurred in fractures of all three roughness levels. During the initial stage of system operation, the seepage velocity in the main fractures was primarily driven by the injection-production pressure difference, leading to a rapid increase in all cases with no significant divergence. In the first five years, the seepage velocity of ScCO_2_ in high-roughness fractures increased sharply. This is attributed to strong local velocity variations caused by high roughness, which raised the seepage pressure within the main fracture, enhanced permeability, and accelerated the development of dominant flow channels. Consequently, ScCO_2_ tended to migrate preferentially through the main fracture under the imposed pressure gradient.

Between years 5 and 10, the seepage pressure in medium-roughness fractures changed markedly, further intensifying non-Darcy flow. As the flow regime evolved, inertial effects became more pronounced, ultimately leading to a higher ScCO_2_ velocity in medium-roughness fractures than in high-roughness fractures by year 10. Although low-roughness fractures exhibit lower macroscopic flow resistance, the better connectivity between main and branch fractures allowed a stable pressure gradient to develop as early as year 2. Moreover, the dispersive effect of branch fractures helped maintain a relatively stable flow state in the main fracture. As a result, the increase in ScCO_2_ seepage velocity in low-roughness fractures slowed after year 2.

Despite experiencing similar volumetric strain during operation, fractures with higher roughness exhibited greater permeability. This is because their rougher surfaces provide more mechanical space for local dilation under the same bulk strain, creating additional connected flow pathways and thereby enhancing effective permeability. Furthermore, as described by Equation 3 in the STAR Methods, fracture permeability is governed by *in-situ* stress, ScCO_2_ seepage pressure, and thermal stress. Under comparable thermal and *in-situ* stress conditions, the significantly higher seepage pressure developed in high-roughness fractures further contributed to the permeability increase.

**Figure 13:**
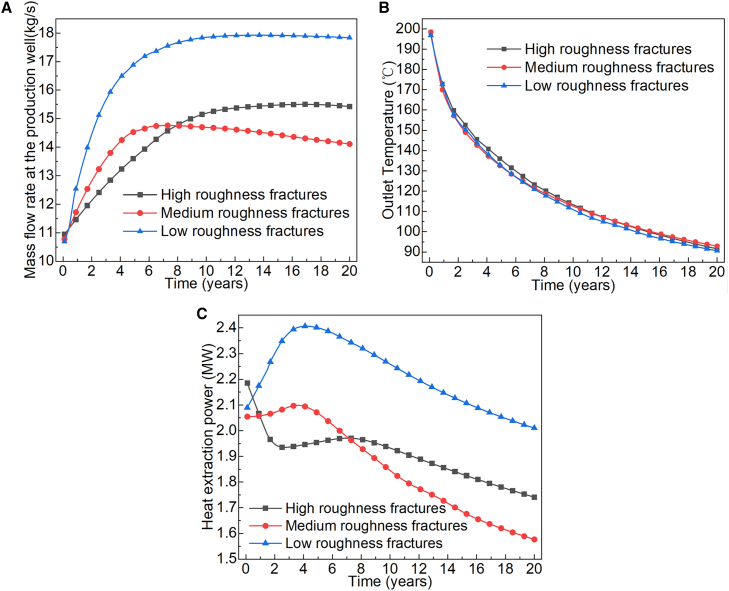
Heat extraction performance of ScCO_2_ in the HDR reservoir with fractures at different *RMS* (A) Mass flow rate at the production well. (B) Outlet temperature. (C) Heat extraction power.

As shown in Figure 13, during system operation, the production temperature of fracture reservoirs with varying roughness levels exhibited similar trends: a rapid decrease in the first 10 years, followed by a gradual decline to the minimum temperature over the next 10 years. However, fractures with low roughness exhibited better ScCO_2_ mass transfer efficiency. Although high and medium roughness fractures had higher Reynolds numbers (Figure 12B) and stronger inertial effects, their macroscopic mass flow rates (Figure 13A) were significantly lower than those of the low roughness fractures. This occurs because the intense vortices and local resistance caused by rough surfaces consume a significant portion of the driving pressure, while more tortuous flow paths reduce the overall transport efficiency. In medium roughness fractures, the higher Reynolds number in the later stage increased flow instability, hindering the sustained development of stable dominant flow channels and aggravating flow resistance and interference. As a result, their mass flow rate became the lowest during the later stage.

In the first two years of operation, the outlet temperature of the three fracture reservoirs decreased by approximately 40°C on average. During the same period, the mass flow rates of ScCO_2_ at the production wells varied by 1.5–2.5 kg s^−1^, directly causing significant differences in heat extraction power. The intensified non-Darcy flow led to a more uneven flow distribution, making the system prone to local short-circuiting and insufficient heat exchange. As the operation continued, these mechanisms ultimately caused differentiated heat extraction efficiencies among the three types of roughness reservoirs.

In summary, the optimal reservoir conditions are achieved with an *RMS* fracture roughness of approximately 0.9413 m and a fracture dip angle of about 30°, where ScCO_2_ exhibits stable flow direction, uniform pressure gradient, and balanced mass transfer.

### Limitations of the study

While this numerical study provides insights into the seepage mechanisms of ScCO_2_ in dynamic rough fractures, several limitations should be acknowledged. First, fracture roughness is characterized primarily by the *RMS* parameter, whereas other important morphological descriptors, such as the *JRC* and fractal dimension, are not explicitly considered. Second, the model neglects multiphase flow behavior and geochemical reactions, which may play a critical role in long-term heat extraction performance and CO_2_-rock interactions. Finally, the findings are based on numerical simulations and lack direct experimental validation under representative pressure-temperature conditions. Addressing these limitations in future work will improve the robustness and predictive capability of the model for field-scale geothermal heat extraction and CO_2_ storage applications.

## Resource availability

### Lead contact

Further information and requests for resources and reagents should be directed to and will be fulfilled by the Lead Contact, Xiao Xiaochun (xxc7902@163.com).

### Materials availability

This study did not generate new unique reagents.

### Data and code availability

• All data reported in this article will be shared by the lead contact upon request.
• This article does not report original code.
• Any additional information required to reanalyze the data reported in this article is available from the lead contact upon request.

## Acknowledgments

The authors gratefully acknowledge the funding support by the 10.13039/501100001809National Natural Science Foundation of China [No.52274203].

## Author contributions

X. X.: conceptualization, formal analysis, supervision, validation, investigation, writing-original draft, writing – review and editing. L. C.: conceptualization, data curation, software, investigation, methodology, writing-original draft, and writing – review and editing. G. P.: software, methodology, visualization, conceptualization, supervision, and writing – original draft.

## Declaration of interests

The authors declare no competing interests.

## STAR★Methods

### Key resources table

**Table:** 

REAGENT or RESOURCE	SOURCE	IDENTIFIER
**Deposited data**		

Rock mechanical properties	Public domain data	References cited

**Software and algorithms**		

COMSOL Multiphysics 6.2	COMSOL AB	https://cn.comsol.com/

### Experimental model and study participant details

#### Numerical reservoir model

This study is based entirely on deterministic numerical simulations of ScCO_2_ flow and heat transfer in a fractured HDR reservoir. No animals, human participants, plants, microorganisms, cell lines, or primary cell cultures were involved.

The reservoir parameters were derived from field measurements of the Matouying geothermal project in Tangshan, Hebei, including burial depth, *in-situ* stress conditions, initial reservoir temperature, and pore pressure. A three-dimensional geometric model was constructed to represent the fractured HDR reservoir system, as described in the main text.

#### Sex and gender considerations

Because this study did not involve human or animal subjects, sex and gender variables were not applicable. Consequently, no sex- or gender-based analyses were performed. This does not affect the generalizability of the numerical findings, as the study focuses exclusively on physical and thermodynamic processes in geological systems.

### Method details

This study uses COMSOL Multiphysics 6.2 software for numerical modeling to simulate the flow and heat transfer behavior of ScCO_2_ in an HDR reservoir model. A single-variable method was employed to investigate the effects of fracture dip angle and surface roughness on heat extraction efficiency. The following provides a detailed description of the experimental design and modeling process.

#### Governing equations

In HDR reservoirs, the coupling effects of the temperature, seepage, and stress fields significantly alter the porosity and permeability of the rock matrix and fractures, with these effects being mutually interactive. In 1974, Louis^13^ summarized the relationship between rock fracture permeability and surface normal stress through rock borehole water pressure tests. Xiao X^31^ derived the evolution law of pore-permeability characteristics in the rock matrix of HDR reservoirs over time under thermo-hydro-mechanical coupling through theoretical derivation. The equations for the variation of porosity and permeability are as follows:

(Equation 1) $$\phi_{S}=1-\left(1-\phi_{S_{0}}\right)\exp\left[-\frac{1}{K_{S}}\left(p-p_{0}\right)+\alpha_{T}\left(T-T_{0}\right)-\left(\varepsilon_{V}-\varepsilon_{V_{0}}\right)\right]$$

(Equation 2) $$k_{S}=k_{S_{0}}{\left\{\frac{1}{\phi_{S_{0}}}-\left(\frac{1}{\phi_{S_{0}}-1}\right)\exp\left[-\frac{1}{K_{S}}\left(p-p_{0}\right)+\alpha_{T}\left(T-T_{0}\right)-\left(\varepsilon_{V}-\varepsilon_{V_{0}}\right)\right]\right\}}^{3}$$

(Equation 3) $$k_{f}=k_{f0}\;\exp\left[-b_{1}\left(\sigma_{n}-ap_{f}\right)\right]$$

where *ϕ*_*S*_ is the rock matrix porosity, dimensionless; *ϕ*_*S*0_ is the initial porosity of the rock matrix, dimensionless; *K*_*S*_ is the bulk modulus of the rock matrix, Pa; *p* is the pore pressure, Pa; *p*_0_ is the initial pore pressure of the reservoir, Pa; *α*_*T*_ is the thermal expansion coefficient of the rock matrix, 1/°C; *T* is the temperature, °C; *T*_0_ is the initial temperature, °C; *ε*_*V*_ is the volumetric strain, dimensionless; *ε*_*V*0_ is the initial volumetric strain, dimensionless; *k*_*S*_ is the permeability of the reservoir rock matrix, m^2^; *k*_*S*0_ is the initial permeability of the rock matrix, m^2^; *k*_*f*_ is the permeability of fractures, m^2^; *k*_*f*0_ is the initial permeability of fracture, m^2^; *α* is the fracture Biot coefficient, dimensionless; *b*_1_ is the normalization constant for fractures, 1/Pa; *p*_*f*_ is the seepage pressure in fractures, Pa; *σ*_*n*_ is the normal stress on fractures, Pa, influenced by thermal stress and *in-situ* stress.

Based on Darcy’s law, the governing equation for the seepage field in the rock matrix is:

(Equation 4) $$\frac{\partial \rho_{L}\phi_{S}}{\partial t}-\nabla \cdot \left(\frac{k_{S}}{\mu }\rho_{L}\nabla p\right)=Q_{H}$$

where *ρ*_*L*_ is the density of ScCO_2_, kg/m^3^; *μ* is the dynamic viscosity of ScCO_2_, Pa·s; ▽*p* is the pressure gradient along the flow path, Pa/m; *Q*_*H*_ is the mass source term of the reservoir matrix seepage field, kg/(m^3^·s).

While Darcy’s law describes linear seepage, it neglects the inertial resistance induced by the flow of a free medium. This inertial effect becomes non-negligible in regions adjacent to porous medium interfaces, such as fractures. Given that fracture permeability typically exceeds that of the reservoir matrix by several orders of magnitude, and considering the substantial hydraulic gradients present at great depths, ScCO_2_ can attain significantly high seepage velocities within these fracture networks. To account for this non-Darcy flow behavior, the governing equation for the fracture seepage field is given by Forchheimer’s modified law,^32^ as follows:

(Equation 5) $$\left\{ \begin{array}{l} d_{f}\frac{\partial }{\partial t}\left(\rho_{L}\phi_{f}\right)+\nabla_{\tau }\cdot \left(d_{f}\rho_{L}u\right)=d_{f}Q_{Hf}\\ -\nabla_{\tau }\cdot p_{f}=\frac{\mu }{k_{f}}u+\beta \rho_{L}\left|u\right|u \end{array}\right.$$

where *ϕ*_*f*_ is the porosity of fractures, dimensionless; *d*_*f*_ is the aperture of fractures, m; *u* is the non-Darcy flow velocity vector, m/s; ∇_*τ*_ is the tangential gradient along the fracture; *β* = *c*_*F*_/(*k*_*f*_)^1/2^ is the Forchheimer coefficient, 1/m; *k*_*f*_ is the permeability of fractures, m^2^; *c*_*F*_ = 1.75/(150*ϕ*_*f*_^3^)^1/2^ is the dimensionless friction coefficient of the fracture; *Q*_*Hf*_ is the mass source term of the fracture seepage field, kg/(m^3^·s).

When the fluid velocity is sufficiently low, the nonlinear inertial term *βρ*_*L*_|***u***|***u*** in Equation 5 becomes negligible compared to the linear viscous term ***u***·*μ*/*k*_*f*_. Under this condition, Equation 5 reduces to the classical Darcy-flow equation, whose solution for an idealized smooth parallel-plate fracture corresponds exactly to the traditional cubic law.

Assuming thermal equilibrium between the reservoir solid and the working fluid, i.e., *T*_*S*_ = *T*_*L*_ = *T*, and since the porosity *ϕ*_*S*_ ≪ 1, it can be approximated that 1 - *ϕ*_*S*_ ≈ 1. By neglecting the influence of thermal radiation, the governing equation for the temperature field of convective heat transfer of ScCO_2_ in the reservoir matrix can be derived based on the fundamental laws of thermodynamics:

(Equation 6) $$\frac{\partial \left[\left(1-\phi_{S}\right)\rho_{S}c_{S}+\phi_{S}\rho_{L}c_{L}\right]\Delta T}{\partial t}=T\alpha_{T}\left(1-\phi_{S}\right)K_{S}\frac{\partial \varepsilon_{V}}{\partial t}-\nabla \cdot \left[\rho_{L}c_{L}\left(\frac{k_{S}}{\mu }\nabla p\right)T\right]+\nabla \cdot \left\{\left[\left(1-\phi_{S}\right)\lambda_{S}+\phi_{S}\lambda_{L}\right]\nabla T\right\}+Q_{T}$$

where *ρ*_*S*_ is the density of the rock matrix, kg/m^3^; *c*_*S*_ is the specific heat capacity of the rock matrix, J/(kg·°C); *c*_*L*_ is the constant-pressure specific heat capacity of ScCO_2_, J/(kg·°C); Δ*T* is the temperature change value, °C; *λ*_*S*_ is the thermal conductivity of the matrix, W/(m·°C); *λ*_*L*_ is the thermal conductivity of ScCO_2_, W/(m·°C); *Q*_*T*_ is the heat source term regulating the thermal balance between the equivalent matrix skeleton and the working fluid in the matrix pores, dimensionless.

The governing equation for the temperature field of convective heat transfer of ScCO_2_ in fractures is:

(Equation 7) $$d_{f}\rho_{S}c_{S}\frac{\partial T}{\partial t}-\nabla_{\tau }\cdot \left[d_{f}\lambda_{f}\nabla_{\tau }T\right]-d_{f}\rho_{L}c_{L}\left(\frac{k_{f}}{\mu }\nabla_{\tau }p_{f}\right)\nabla_{\tau }T=d_{f}Q_{T}$$

where *λ*_*f*_ = *λ*_*S*_ is the fracture thermal conductivity, W/(m·°C).

Under the thermo-hydro-mechanical coupling effect, the deformation of the reservoir is primarily caused by three factors: thermal strain due to temperature changes, matrix skeleton deformation caused by pore pressure variations from working fluid seepage, and strain induced by *in-situ* stress. Therefore, the governing equation for the reservoir stress field is:

(Equation 8) $$Gu_{i,j}+\left(\lambda +G\right)u_{j,ji}-\alpha_{B}p_{i}-\alpha_{T}KT_{i}+F_{i}=0$$

where *G* is the shear modulus of the rock matrix, Pa; *u*_*i*,*j*_, *u*_*j*,*ji*_ are the reservoir strain components, dimensionless; *λ* is Lamé’s first parameter, Pa; *α*_*B*_ is the Biot coefficient of the reservoir matrix, dimensionless; *p*_*i*_ is the pore pressure component of the reservoir, Pa; *T*_*i*_ is the temperature component of the reservoir, °C; *F*_*i*_ is the body force component acting on the reservoir, Pa. Where: *G* = *E*/[2(1+*υ*)]; *λ* = *Eυ*/[(1+*υ*)(1-2*υ*)]; *α*_*B*_ = 1 - *K*/*K*_*S*_; *E* is the elastic modulus of the rock matrix, Pa; *υ* is the Poisson’s ratio of the rock matrix, dimensionless; *K* is the bulk modulus of the rock skeleton, Pa. The third term on the left side of the equation represents the influence of working fluid seepage on reservoir deformation, and the fourth term on the left side represents the influence of temperature changes on reservoir deformation.

#### Thermophysical property parameters of ScCO_2_

Upon transitioning to a supercritical state, carbon dioxide exhibits significant alterations in its thermophysical properties. Owing to the extended operational time frame of EGS and the inherent ability of the deep subsurface to sustain prolonged high-temperature and high-pressure environments, this study excludes consideration of the phase transition process leading to the supercritical state of CO_2_. Consequently, to enhance simulation accuracy, data fitting procedures were implemented, which also accommodated an appropriate increase in the model’s nonlinearity. The corresponding thermophysical property expressions for ScCO_2_ are presented below.^33^^,^^34^

(Equation 9) $$\mu \left(T,p\right)=\left(7.14\times 10-9T^{2}+5.642\times 10-6T-5.71\times 10-9p^{2}+2.186\times 10-6p+0.0011\right)$$

(Equation 10) $$c_{L}\left(T,p\right)=\left(-4.9\times 10-5T^{3}+0.084T^{2}-49.11T-0.47p^{3}-42.1p^{2}+1200p+276.3\right)$$

(Equation 11) $$\rho_{L}\left(T,p\right)=\left(3.6\times 10-4T^{2}-0.3693T^{2}+122T-0.333p^{3}+32.54p-12720\right)$$

(Equation 12) $$\lambda_{L}\left(T,p\right)=\left(\begin{array}{l} -1.75\times 10^{-8}T^{3}+2.29\times 10^{-5}T^{2}-10^{-2}T\\ -1.89\times 10^{-5}p^{3}+7\times 10^{-4}p^{2}-6\times 10^{-3}p+1.46 \end{array}\right)$$

#### Model construction

##### Data construction of three-dimensional rough fracture surfaces

To reproduce the irregular and random characteristics of natural HDR fractures, rough surfaces were generated using a spectral superposition method. Fracture surface height fields were constructed by superimposing cosine functions with different spatial frequencies. Random components following normal and uniform probability distributions were introduced to represent natural variability. The governing equations for the spatial height field are given as follows:

(Equation 13) $$\left\{ \begin{array}{cc} x=s_{1}\text{;} & y=s_{2}\\ z=0.5\times & \sum_{\begin{array}{l} m,n=-N\\ \left(m,n\right)\neq \left(0,0\right) \end{array}}^{N}\frac{g\left(m,n\right)}{\left(m^{2}+n^{2}\right)\frac{b}{2}}\cos\left[2\pi \left(ms_{1}+n_{1}\right)+u\left(m,n\right)\right] \end{array}\right.$$

where *s*_1_ and *s*_2_ are the spatial coordinates of *x* and *y*; (-*N*, *N*) is the spatial resolution, representing the range of spatial frequencies; *b* is the spectral exponent, indicating the attenuation of high frequencies; 0.5 is the global amplitude scaling factor; (*m*, *n*) are the spatial frequency components; *g*(*m*, *n*) is the amplitude modulation function; *u*(*m*, *n*) is the phase modulation function.

*s*_1_ and *s*_2_ control the extension of the rough fracture surface along the *x*-axis and *y* axis directions. *N* controls the number of high-frequency components; increasing the value of *N* allows the equation to incorporate more high-frequency components, adding small-scale details to the fracture surface, thereby making it rougher. The *b*-value controls the magnitude of the high-frequency components; increasing the *b*-value weakens the contribution of high-frequency components, making the fracture surface smoother.

##### Characterization of roughness

The calculation of the *RMS* for the three-dimensional rough fracture surface is implemented using MATLAB programming, with the following steps.

• Spatially discretize *s*_1_ and *s*_2_, with the number of nodes *N* set to 100 in each direction, resulting in a total of 10,000 nodes;
• Retrieve the height data *z*(*s*_1*i*_, *s*_2*j*_) for each node and store it in a two-dimensional matrix (100 rows, 100 columns), then convert the matrix into a column vector;
• Sum the heights of all data points: $Z=z_{1}+\cdot \cdot \cdot +z_{10000}$;
• Calculate the average height: $Z_{a}=Z/10000$;
• Compute the squared deviation of each point’s height relative to the average height: ${\left(\Delta z_{n}\right)}^{2}={\left(z_{n}-Z_{a}\right)}^{2}$;
• Calculate the mean of the sum of squared deviations for all points: $A=1/10000\left[{\left(\Delta z_{1}\right)}^{2}+\cdot \cdot \cdot +{\left(\Delta z_{10000}\right)}^{2}\right]$;
• Calculate the *RMS* value: $RMS=\sqrt{A}$.

The *RMS* values calculated for high-frequency attenuation indices (*b*-values) of 1.4, 1.6, and 1.8 are 1.1093 m, 1.0043 m, and 0.9413 m, respectively, which correspond to high-roughness fractures, medium-roughness fractures, and low-roughness fractures.

##### Establishment of the geometric model

Assuming that the HDR reservoir is buried at a depth of 4000 m, with an initial reservoir temperature of 200 °C, the geometric model of the reservoir is a rectangular cuboid with dimensions of 200 m × 200 m × 130 m. The wellbore diameter is 0.26 m, and the spacing between the injection and production wells is 50 m. The blue cylinder represents the injection well, and the red cylinder represents the production well, with the fracture located between the two wells. Specified displacements are applied to the outer boundaries of the model: boundaries parallel to the *xy*-plane are constrained to displace only in the *z*-direction, boundaries parallel to the *yz*-plane are constrained to displace only in the *x*-direction, and boundaries parallel to the *zx*-plane are constrained to displace only in the *y*-direction. The outer boundaries of the model are no-flow boundaries, and the surrounding sides are set as open boundaries with a temperature of 200 °C. The specific parameter values of the model are shown in Table 1. The initial matrix pore pressure *p*_0_ and the *in-situ* stresses (100 MPa in the z-direction, and 120 MPa in both the *x*- and *y*-directions) are derived from the measured data of the Matouying geothermal project in Tangshan, Hebei.^30^

The following provides a detailed description of the modeling process.

• Fracture Dip Angle: By selecting the angle between the baseline of the rough fracture and the line connecting the injection and production wells as a variable, a single-fracture heat extraction model for the HDR reservoir with different dip angles was established. Three geometric models of the reservoir with dip angles *θ* of 0°, 30°, and 60° were created, and injection well and production well penetrated the rough fracture surface in all models. The fracture surface is a medium-roughness surface with an *RMS* value of 1.0043 m when *b* = 1.6, and the “length × width” of the fracture surface is 50 m × 50 m.
• Fracture Roughness: Based on the Monte Carlo method, generate five fractures with dip angles ranging from 0° to 60° within a rectangular prism region of dimensions 200 m × 200 m × 100m. By keeping the fundamental parameters of the HDR reservoir geometric model constant and varying the *b* value to 1.4, 1.6, and 1.8, HDR reservoirs with different roughness crack surfaces can be obtained, yielding *RMS* values of 1.1093 m, 1.0043 m, and 0.9413 m, respectively. Fracture surfaces with high, medium, and low roughness were generated using the aforementioned method, and the impact of these roughness levels on the flow and heat exchange of ScCO_2_ was simulated.

#### Model verification

Enhanced geothermal systems involve complex coupling problems of mutual influence and constraints among the temperature, seepage, and stress fields. Therefore, after establishing the THM coupled governing equations, it is necessary to validate them. This paper adopts the method of comparing the analytical solution for the flow and heat transfer problem of a single fracture in a two-dimensional infinite rock mass with the numerical solution of the simplified THM coupled model to verify the accuracy of the THM model presented herein.

In the two-dimensional single fracture model, the rock matrix is assumed to have infinite extent, and the fracture extends infinitely in the horizontal direction. As shown in Figure 4A, the initial temperature of the entire rock is assumed to be *T*_0_, with the heat exchange working fluid injected from the left side of the fracture at an injection velocity of *u*_in_ and a constant injection temperature of *T*_in_. The working fluid flows through the fracture and extracts heat from the rock matrix. The basic parameters are provided in Table 2. The temperature distribution in the fracture is calculated using the analytical solution proposed by Barends,^35^ as shown in Equation 14. COMSOL Multiphysics 6.2 is then used to construct a THM coupled numerical model for numerical solving. The comparison between the numerical and analytical temperature solutions at different monitoring points is shown in Figure 4B. As seen in the figure, the numerical solution for temperature aligns closely with the analytical solution, with a small relative error between them. Therefore, it can be concluded that the accuracy of the THM coupled governing equations presented in this paper is reliable and suitable for the subsequent work in this study.

(Equation 14) $$T_{x}=T_{i}+\left(T_{\text{in}}-T_{i}\right)\text{erfc}\left(\frac{\lambda_{S}x/\left(\rho_{w}c_{w}d_{fr}\right)}{\sqrt{u_{\text{in}}\left(u_{\text{in}}t-x\right)\lambda_{S}/\left(\rho_{R}c_{R}\right)}}\right)U\left(t-\frac{x}{u_{\text{in}}}\right)$$

where *T*_*x*_ is the temperature at coordinate *x* in the fracture, °C; *T*_i_ is the initial temperature of the rock matrix and the fracture, °C; *T*_in_ is the fluid mass injection temperature, °C; *λ*_*S*_ is the thermal conductivity, W/(m·°C); *x* is the horizontal coordinate, m; *ρ*_*w*_ is the density of the fluid mass, kg/m^3^; *c*_*w*_ is the isobaric specific heat capacity of the fluid mass, J/(kg·°C);*d*_*fr*_ is the fracture opening, m. *t* is the injection time, s; *ρ*_*R*_ is the density of rock, kg/m^3^; *c*_*R*_ is the isobaric specific heat capacity of rock, J/(kg·°C); erfc is an unfounded complementary error function, unfounded; *U* is an unfounded unit step function, unfounded.

#### Replicates and statistical considerations

This study is based on deterministic numerical simulations. No biological replicates, randomization procedures, blinding, or inferential statistical analyses were applicable. All simulation scenarios were predefined and executed under controlled numerical conditions. No data were excluded from analysis.

### Quantification and statistical analysis

As this study is based on deterministic numerical simulations, no experimental data collection, randomization procedures, or inferential statistical analyses were involved. The results were evaluated through quantitative analysis of the simulation outputs and direct comparisons among different modeling scenarios.

All data were generated using COMSOL Multiphysics 6.2. The simulated physical quantities were post-processed to obtain the parameters required for analysis. Among these, the Reynolds number and outlet heat extraction power were calculated as follows:

The Reynolds number quantitatively characterizes the relative influence of inertial and viscous forces in fluid flow through fractures, thereby determining the flow regime of the heat transfer medium. A low Reynolds number (*Re* < 10) indicates laminar flow, where linear Darcy’s law is applicable. At high Reynolds numbers (*Re* > 10), the inertial effect becomes dominant, leading to non-Darcy flow behavior. The magnitude of the Reynolds number can be used to assess the extent of non-Darcy flow. The Reynolds number is calculated as follows:

(Equation 15) $$Re=\frac{r_{L}\times u_{L}\times d_{f}}{m}$$

where ***u***_*L*_ is the flow velocity of ScCO_2_, m/s.

The heat extraction rate from production wells is a critical quantitative metric for assessing the efficiency of EGS in exploiting HDR resources. It directly illustrates how variables, such as working fluids, injection conditions, and reservoir characteristics, differentially impact the heat extraction performance of EGS.^36^ The formula for calculating the heat extraction rate from production wells is as follows:

(Equation 16) $$P_{pro}=q_{pro}\rho_{L}c_{L}\left(T_{pro}-T_{inj}\right)$$

where *P*_pro_ is the heat extraction power of the production well, MW; *q*_pro_ is the mass flow rate of ScCO_2_ at the outlet of the production well, kg/s; *T*_pro_ is the temperature of the produced fluid, °C; *T*_inj_ is the temperature of the injected fluid, °C.
